# Genetic diversity and phylogeography of the endemic species *Chimonobambusa utilis* growing in southwest China: Chloroplast DNA sequence and microsatellite marker analyses

**DOI:** 10.3389/fpls.2022.943225

**Published:** 2022-11-03

**Authors:** Yanjiang Liu, Mingli Wu, Xue Xu, Xiao Zhu, Zhaoxia Dai, Guangqian Gou

**Affiliations:** ^1^ Key laboratory of Plant Resource Conservation and Germplasm Innovation in Mountainous Region (Ministry of Education), College of Life Sciences/Institute of Agro-Bioengineering, Guizhou University, Guiyang, China; ^2^ Bamboo Research Institute, Guizhou University, Guiyang, China; ^3^ College of Forestry, Guizhou University, Guiyang, China

**Keywords:** *chimonobambusa utilis*, genetic diversity, endemic plant, historical dynamics, haplotype

## Abstract

*Chimonobambusa utilis* (Keng) Keng F is an endemic species distributed only in the Daluoshan Mountains, southwest China. *Ch. utilis* is popular due to its unique flavor and deliciousness and plays an important role in the industrial revolution in many counties in China. A total of 20 natural populations were sampled from the entire distribution range of *Ch. utilis*. In the present study, we used five EST-SSR molecular markers, three chloroplast DNA (*trn*H-*psb*A, *atp*F-*atp*H, and *psb*K-*psb*I), and one ITS molecular marker to elucidate the genetic diversity and phylogeography analyses of these *Ch. utilis* populations. The results exhibited that *Ch. utilis* populations showed lower genetic diversity than other angiosperms (*H_T_* = 0.752, *H_S_* = 0.364, and *F_ST_* = 0.05021 for EST-SSR; *H_T_* = 0.956, *Hs* = 0.507, and *F_ST_* = 0.70121 for cpDNA; *H_T_* = 0.868, *Hs* = 0.495, and *F_ST_* = 0.70121 for nrDNA). A total of 40 alleles were detected for five polymorphic loci. We detected 20 polymorphic sites and 11 haplotypes within 1,398 bp of cpDNA and 59 polymorphic sites and 32 haplotypes within the 589 bp of the ITS sequence. Based on the haplotype distribution, we infer that there were at least two glacial refuges of *Ch. utilis* populations during the Quaternary Ice Age. The genetic and geographic distance were correlated (*p* < 0.05), indicating that narrow distribution might be the primary cause of the low genetic differentiation of *Ch. utilis* populations. Based on the genetic diversity of *Ch. utilis* populations, we recommend implementing effective genetic resource management and sustainable utilization.

## Introduction

*Chimonobambusa utilis*, a member of the Poaceae family *Chimonobambusa*, is an endemic species of southwest China and is widely distributed in the Daloushan Mountain area within an altitude range of 1,000 to 2,300 m. It has strong soil adaptability with pH 4.0–8.0, such as black forest soil, nursery loam, and even karst environment (Lou, 2021). The bamboo shoots of *Ch. utilis* can be eaten fresh or processed into dried shoots, flavor snacks, or canned foods. They have become a favorite food in people’s daily lives and are also used in medicine, healthcare, feeding, and other functions (Liu et al., 2013; Li et al., 2015; Liu et al., 2018). In addition, because of its strong leaves, moderate number of leaves, and colorful shoots, the transition period from bamboo shoots to bamboo can be as long as half a year, and a bamboo forest is quiet and pleasant, thus having high ornamental value (Lou et al., 2021). Growing bamboo for its economic value has become popular in southwest China, where it plays an essential role in establishing urban ecological landscapes and the growth of the tourist industry. At present, the main research direction is to study the germplasm resources, growth dynamics, cultivation, and nutritional components of *Ch. utilis* (Tang et al., 2019; Ren et al., 2021; Zhu et al., 2021), but there are few reports on the population of *Ch. utilis* at the molecular level. Due to the distinctive features of bamboo, the flowering cycle is long and complicated, and environmental changes frequently make it challenging to identify the reproductive and nutritional traits that can be observed morphologically. As a consequence, systematic study findings based on the approach of categorization that simply considers morphological identification are often questioned and may not be reliable (Das et al., 2007). With the advancement of molecular biology and development of sequencing technology, researchers are constantly studying the genetic diversity, species formation, and genetic differentiation of plants (Li et al., 2019; Chen et al., 2021; Djedid et al., 2022). Therefore, we can rely on plant DNA molecular technology to distinguish different germplasm and study the population genetic differentiation, genetic structure, and pedigree geography of bamboo species.

The topography of mountainous areas in southwest China is characterized by a mountainous landscape and geomorphic features (Xie et al., 2017; Du et al., 2019). This region is considered as one of the richest and most diverse biodiversity hotspots. (Kang et al., 2014; Tian and Wariss, 2021). Historical orogeny and climate change will lead to the habitat fragmentation of species, which will split a habitat into multiple plate populations, which may lead to high gene flow and gene drift between or within populations, and eventually, alien differentiation will occur and form new species. Genetic diversity plays an important role in protecting genetic diversity, which is the basis of ecosystem diversity and species diversity and the premise of adaptation, development, and evolution of species in the process of evolution (Shen et al., 2022). The spatial distribution pattern of species determines the genetic diversity of species, historical evolution, geological climate change, and other factors (Ren et al., 2017; Zhao et al., 2021), and its size determines the ability of a population or species to open up a new environment and adapt to environmental changes. It can further analyze its evolutionary potential and provide important data support for future development (Yu et al., 2020).

Therefore, the genetic background of *Ch. utilis* was studied using molecular markers. The level of genetic diversity and genetic structure of the natural population in *Ch. utilis* was revealed, the population dynamics and distribution pattern were discussed, and the excellent provenance population was selected, providing a theoretical basis for auxiliary breeding, construction of DNA fingerprinting approaches, and exploitation and utilization of seed resources. This study aims to provide strategic help for the rational planting, cultivation, and management of *Ch. utilis* and to provide a reference for studies addressing the effects of complex geographical environments and climate fluctuations on the genetic differentiation and distribution pattern of species in southwest China.

Here, after a comprehensive investigation and extensive sampling of the natural population of *Ch. utilis*, molecular markers with different genetic backgrounds and evolution rates were combined for the first time using five EST-SSR markers and three cpDNA regions (*trn*A-*trn*H, *atp*F-*atp*H, and *psb*K-*psb*I) and one ITS sequence to make the results more comprehensive and reliable. We expect to find the causes of the genetic variation by studying the genetic diversity and phylogenetic geography of *Ch. utilis*, and based on the level of genetic diversity and genetic differentiation of *Ch. utilis*. Finally, based on the discussion of various conclusions, put forward the management strategy of scientific and feasible protection measures, and provide a certain scientific theoretical basis for the protection, development, and utilization of the *Ch. utilis* germplasm resources.

## Materials and methods

### Plant sampling

**Figure 1:**
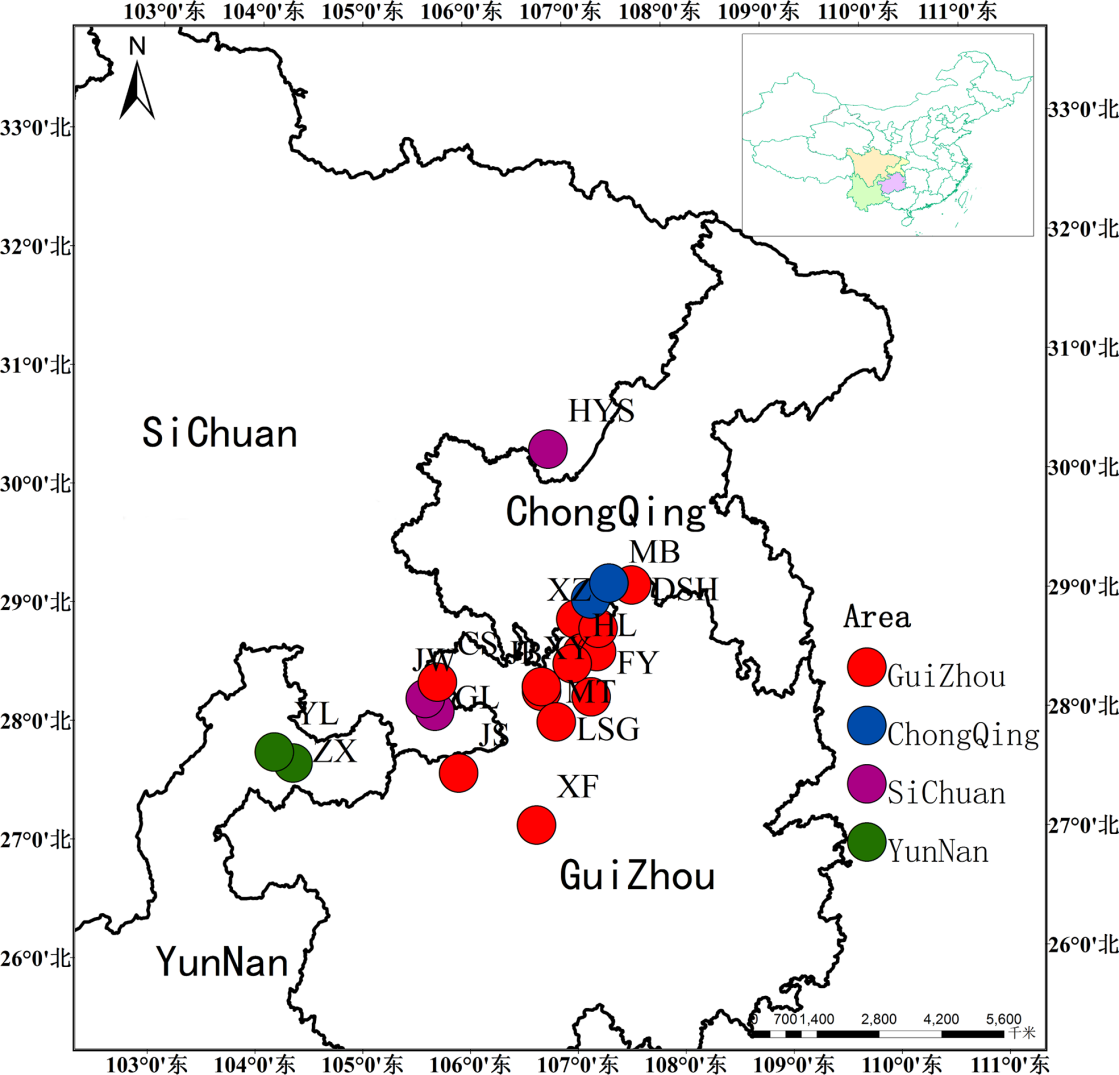
Sampling distribution of *Ch. utilis* in southwest China.

We have collected more than 400 natural individuals of *Ch. utilis* from 20 populations, which presents its entire geographical distribution. Twenty to twenty-five individuals were collected from each population, and each individual was 45 m apart (Chen, 2018). Fresh, tender leaves without obvious diseases and insect pests were collected by GPS software and dried with silica gel for total DNA extraction (**Figure 1**, **Table 1**). Among them, 14 representative populations and 140 individuals were used for EST-SSR molecular marker selection (**Supplementary Table 1**). All specimens were stored in the Natural Museum of Guizhou University (accession number: GACP), Guizhou University, China.

**Table 1:** Sampling information of *Ch. utilis*.

ID	Location	Individuals (*n*)	Latitude (N)	Longitude (E)	Altitude (m)
KKS	Kuankuoshui, GuiZhou	20	107.17	28.20	1,433
JB	Jiuba, GuiZhou	20	106.70	28.86	1,354
QB	Qingba, GuiZhou	20	107.04	28.26	1,781
XF	Fengcisi, GuiZhou	20	106.63	28.30	1,431
JS	Taiping, GuiZhou	20	105.90	27.13	1,324.43
MT	Miaotang, GuiZhou	20	107.09	29.14	1,577
XY	Xianyuan, GuiZhou	20	106.70	28.78	1,633.75
LSG	Loushanguan, GuiZhou	20	106.83	28.58	1,724.86
DSH	Dashahe, GuiZhou	20	107.58	28.57	1,351.06
FY	Fuyan, GuiZhou	20	107.23	28.00	1,544.97
XZ	XinZhou, GuiZhou	20	107.25	28.78	1,394.94
HL	Cuanglian, GuiZhou	20	106.99	28.49	1,715.13
GFD	Gufodong, ChongQing	20	107.18	29.03	2,074.47
HYS	Huayingshan, SiChuan	20	106.79	30.30	1,972.11
MB	Miaoba, ChongQing	21	107.36	29.16	1,348.62
GL	Gulin, SiChuan	20	105.68	28.10	1,533.77
JW	Xuyong, SiChuan	20	105.59	28.21	1,320.04
ZX	Zhenxiong, YunNan	25	104.33	27.66	1,799.96
YL	Yiliang, YunNan	22	104.16	27.75	1,832.11
CS	Liangkouhe, GuiZhou	22	105.71	28.35	1,260.49

### DNA extraction and procedures

DNA was extracted from dried leaves using the Plant Genomic DNA Kit (Tiangen Biotech, Beijing, China). DNA quality was tested using 1.5% agarose gel electrophoresis. The amount of DNA was estimated by spectrophotometry (Meipuda, Shanghai, China). Then, dilution was performed by standardizing all samples to 20 ng DNA/μl and storing it at −80°C.

Microsatellite markers were selected from previously optimized markers in our laboratory and the published literature for screening (Li, 2008; Zhu et al., 2021). Internal transcribed spacer (ITS) sequences and cpDNA fragments (*trn*H-*psb*A, *atp*F-*atp*H, and *psb*K-*psb*I) were amplified (Cai et al., 2012; **Supplementary Table 2**).

The PCR amplification of cpDNA and ITS regions were performed in 17-μl reaction volumes containing 7 μl of 2× Taq PCR Master Mix (Tiangen Biotech, Beijing, China), 0.5 μl of each primer, 1 μl of template DNA, and 8 μl of double-distilled water. The reactions were conducted with the following conditions: initial 2-min denaturation at 95°C followed by 30 cycles of 1-min denaturation at 95°C, 55°C for 10 s with a ramp of 0.3°C/s to 65°C, and 65°C for 1 min, with a final extension at 65°C for 10 min. Agarose gel electrophoresis detection of all PCR products showed clear bands without the occurrence of the indistinct band; obvious primer dimmer and tailing phenomena were directly purified and sequenced. The purified PCR products were sequenced in an ABI 3730 (Thermo Fisher Scientific). The results of chloroplast DNA sequences and nuclear fragments were checked using Chromas 2.6, then manually aligned employing CLUSTAL W in MEGA 6 and adjusted (Tamura et al., 2013). All sequence results were deposited in the GenBank database under accession numbers (ON211020–ON211030 for cpDNA and ON206010–ON206041 for ITS).

Twenty pairs of EST-SSR primers were selected, from which five pairs of EST-SSR primers were successfully amplified with high polymorphism and homology (**Supplementary Table 3**). The volume of the PCR mix was 25 μl: 12.5 μl 2× Taq PCR Master Mix, the upstream and downstream primers (1 μl each), 1 μl of DNA template, and 9.5 μl of ddH_2_O. The reaction parameters of PCR amplifications were as follows: pre-denaturation at 94°C for 3 min, denaturation at 94°C for 30 s, annealing at 55–60.2°C for 30 s, extension at 72°C for 1 min, a total of 30 cycles, and extension at 72°C for 5 min. The selected primer pairs were labeled with a fluorescent dye (FAM) at the 5′ end, and PCR products were analyzed using an ABI3730.

### cpDNA and ITS sequences

Sequences from three cpDNA fragments and ITS sequences were concatenated into a sequence by the software PhyloSuite v1.1.15 (Zhang et al., 2019). The DnaSP version 5.0 (Librado and Rozas, 2009) was used to identify different haplotypes (n), haplotype diversity (*Hd*), nucleotide diversity (*Pi*), and the number of polymorphic sites (π). Population differentiation indices (*Gst* and *Nst*) were calculated using the program PERMUT with 1,000 permutations (Pons and Petit, 1996). The genealogical relationships among all the haplotypes were performed using a median-joining method in Network v.5 (Bandelt et al., 1999; http://www.fluxus-engineering.com/sharenet.htm). The geographical distribution of populations and haplotypes was mapped in ArcGIS v.10.2.

Average genetic diversity within populations (*HS*), total genetic diversity (*HT*), and the genetic variation, differentiation, and pairwise fixation indices (*F_ST_*) within and among populations were statistically tested through analyses of molecular variance (AMOVAs) in ARELQUIN v3.1.16 (https://www.fousoft.com/arlequin.html). To better understand the population growth and expansion of cpDNA and nrDNA fragments of *Ch. utilis*, both neutrality tests and mismatch analysis were implemented in DnaSP v.5.

### EST-SSR analysis

Peak analysis was performed with Gene Mapper 4.1 software, the signal value of the peak was above 200 bp, and the peak shapes are similar at the same site. For each microsatellite locus, the expected heterozygosity (*He*), observed heterozygosity (*Ho*), effective numbers of alleles (*Ne*), the mean number of alleles (*Na*), percentage of polymorphic loci (PPB), and Wright’s *F* statistics parameters (*F_IS_*, *F_IT_*, and *F_ST_*) were analyzed using GenAIEx6.5 (Peakall and Smouse, 2012). The polymorphism information content (PIC) is one of the important indexes to measure the polymorphism of SSR locus using CERVUS 3.0 (Kalinowski et al., 2007). The cluster analysis was carried out to characterize population structure in STRUCTURE version 2.3 (Pritchard et al., 2009). The parameter settings of about 20 independent simulations were run for a *K*-value of 2–15 with 10,000 length of burnin period and a number of MCMC reps after burnin of 100,000. Structure harvester was then utilized to estimate the most likely number (*K*) (Earl, 2012; http://taylor0.biology.ucla.edu/structureHarvester/) of genetic clusters based on both log-likelihood L (*K*) and Delta *K* (Δ*K*) (Evanno et al., 2005) methods. Principal coordinate analysis (PCoA) and Mantel tests were carried out using GenAIEx6.5 based on Nei’s genetic distance for EST-SSR data. An AMOVA among and within populations was carried out using the program Arlequin version 3.5 to ensure the accuracy of the estimation of variance components (Excoffier and Lischer, 2010).

PopGene32 software (Yeh et al., 1997) was used to calculate Nei’s genetic distance of 14 populations of *Ch. utilis*. The software was then used to detect correlations between genetic distance and geographical distance, genetic distance, and population altitude. Bottleneck 1.2.02 (Cornuet and Luikart, 1996) was used to test for recent bottleneck events in the species. The demographic history of populations was explored under the stepwise mutation model (SMM) and two-phase model (TPM) and by a heterozygosity excess test. Computation was performed using two methods (Sign and Wilcoxon tests) appropriate for a population number < 20.

## Results

### cpDNA and ITS sequences analysis

**Figure 2:**
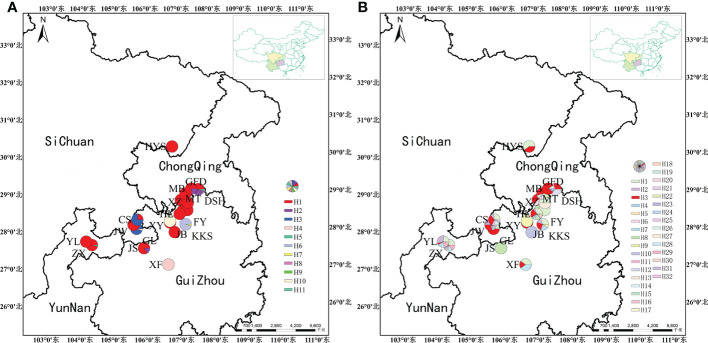
Geographic distribution of haplotype based on cpDNA **(A)** and nrDNA **(B)** sequences.

We identified the cpDNA consensus sequences from 410 individuals in 20 populations with a total of 1,398 bp in length containing 20 polymorphic sites (**Supplementary Table 4**), and the total GC content was 34.68%. We found 11 chloroplast haplotypes (H1–H11); among them, H1 was the most common, occurring in 296 individuals with a wide distribution in 16 populations, suggesting that it is the most ancient haplotype. Haplotypes H4–H11 were unique haplotypes. Haplotype H4 was unique to the GL population; haplotype H5 was unique to the ZX population. Haplotypes H6 and H7 appeared in the KKS population (**Figure 2A**). The total nucleotide diversity (*Pi*) of all populations was 0.00038, with most populations having a null value, and the average was 0.00013. Total haplotype diversity (*Hd*) was 0.4641, and the average was 0.4343 (**Table 2**). It is noteworthy that the DSH population had the highest cpDNA nucleotide and haplotype diversity in *Ch. utilis* (0.000760 and 0.511, respectively).

**Table 2:** Distribution and genetic diversity parameters of cpDNA haplotypes in *Ch. utilis*.

No.	Population	cpDNA					No.	Population	cpDNA				
		*N*	*Nh*	π (×10^−3^)	*H_d_*	Haplotypes (*N*)			*N*	*Nh*	π (×10^−3^)	*H_d_*	Haplotypes (*N*)
1	KKS	20	2	0.070	0.100	**H6 (19)**, **H7 (1)**	11	XZ	20	1	0	0	H1 (20)
2	JB	20	2	0.070	0.100	H1 (19), **H8 (1)**	12	HL	20	1	0	0	H1 (20)
3	QB	20	1	0	0	H1 (20)	13	GFD	20	1	0	0	H1 (20)
4	XF	20	1	0	0	**H9 (20)**	14	HYS	20	1	0	0	H1 (20)
5	JS	20	2	0. 390	0.268	H1 (17), H2 (3)	15	MB	21	2	0. 550	0.381	H1 (16), H2 (5)
6	MT	20	1	0	0	H1 (20)	16	GL	20	2	0.430	0.100	H3 (19), **H4 (1)**
7	XY	20	1	0	0	**H10 (20)**	17	JW	20	1	0	0	H1 (20)
8	LSG	20	1	0	0	H1 (20)	18	ZX	25	2	0.064	0.080	H1 (24), **H5 (1)**
9	DSH	20	3	0. 760	0.511	H1 (13), H2 (6), **H11 (1)**	19	YL	22	1	0	0	H1 (22)
10	FY	20	1	0	0	H1 (20)	20	CS	22	2	0.260	0.368	H1 (5), H3 (17)

Bold represents the unique haplotype, N represents the number of individuals, Nh represents the number of haplotypes, π represents the nucleotide diversity, and H_d_ represents the haplotype diversity.

For ITS data, the total length was 589 bp, including 59 polymorphic sites, and the content of GC was 70.29%. A total of 32 haplotypes (H1–H32) were identified based on nrDNA fragments. Among them, haplotype H3 contains 105 individuals with the highest frequency and the widest range of distribution, and we speculate that this is an ancient haplotype. At the species level, using ITS data, the *Pi* value was 6.13 × 10^−3^, with an average value was 1.8325 × 10^−3^. The *Hd* value was 0.841, with an average value of 0.4343 (**Table 3**; **Figure 2B**).

**Table 3:** Distribution and genetic diversity parameters of nrDNA haplotypes in *Ch. utilis*.

No.	Population	nrDNA				
		*N*	*Nh*	π (×10^−3^)	*H_d_*	Haplotypes (*N*)
1	KKS	20	5	2.350	0.795	H1 (6), H2 (3), H3 (5), H4 (4), H5 (2)
2	JB	20	3	0.820	0.405	H1 (4), H2 (1), H3 (15)
3	QB	20	3	1.150	0.595	H1 (2), H3 (10), H6 (8)
4	XF	20	3	0.560	0.328	H1 (8), H3 (4), H7 (8)
5	JS	20	1	0	0	H8 (20)
6	MT	20	1	0	0	H8 (20)
7	XY	20	1	0	0	**H9 (20)**
8	LSG	20	1	0	0	**H10 (20)**
9	DSH	20	3	1.500	0.695	H3 (6), **H11 (6)**, **H12 (8)**
10	FY	20	1	0	0	H1 (20)
11	XZ	20	1	0	0	**H13 (20)**
12	HL	20	9	4.030	0.853	H1 (6), H2 (1), H3 (5), H4 (1), **H14 (1)**, **H15 (3)**, **H16 (1), H17 **(1), **H18** (1)
13	GFD	20	5	15.670	0.668	H3 (11), **H19(2)**, **H20 (1)**, **H21 (2)**, **H22 (4)**
14	HYS	20	2	0.860	0.505	H1 (12), H3 (8)
15	MB	21	4	1.410	0.348	H1 (2), H3 (17), **H23 (1)**, **H24 (1)**
16	GL	20	2	0.890	0.526	H1 (10), H3 (10)
17	JW	20	7	1.720	0.726	H1 (4), H2 (6), H3 (6), H4 (1), H7 (1), H25 (1), H26 (1)
18	ZX	25	7	1.730	0.757	H1 (8), H2 (7), H7 (1), H25 (1), **H27 (1)**, H28 (3), H29 (4)
19	YL	22	7	1.800	0.771	H1 (5), H2 (7), H3 (1), H7 (2), H28 (2), H29 (4), **H30 (1)**
20	CS	22	7	2.160	0.714	H1 (6), H2 (2), H3(7), H25 (2), H26 (3), **H31 (1), H32 (1)**

Bold black represents the unique haplotype, N represents the number of individuals, Nh represents the number of haplotypes, π represents the nucleotide diversity, Hd represents the Haplotype diversity.

The AMOVA based on the cpDNA and ITS sequences data further revealed the genetic structure from populations of *Ch. utilis*. It is apparent that a higher genetic variation was observed between populations (84.14%, *p* < 0.001) than within populations (15.86%, *p* < 0.001) at the cpDNA level. The *F_ST_* value was 0.84135, which also indicates a significant level. The ITS sequence shows the same result, similar to those of cpDNA, the variation came from among populations, and the *F_ST_* value was 0.84135 (**Table 4**).

**Table 4:** AMOVA analysis based on the cpDNA and nrDNA for *Ch. utilis* populations.

Source of variation	cpDNA					nrDNA				
	d.f.	Sum of squares	Variance components	Percentage of variation	Fixation Index	d.f.	Sum of squares	Variance components	Percentage of variation	Fixation Index
Among populations	19	188.078	0.47856	84.14	0.84135	19	611.693	1.53875	70.12	0.70121
Within populations	390	35.193	0.09024	15.86		390	255.709	0.65566	29.88	
Total	409	223.271	0.56879			409	867.402	2.19442		

Genetic structure analysis of *Ch. utilis* (based on cpDNA and ITS sequences) was done using Permut v.2.0 (**Table 5**). It is apparent from this table that the total genetic (*H_T_*) and the average within-population diversity (*H_S_*) were 0.956 and 0.507, respectively, based on combined cpDNA sequences. The population genetic differentiation coefficients (*G_ST_* = 0.470, *N_ST_* = 0.976, *N_ST_* > *G_ST_*, *p* < 0.05) showed a significant pedigree geographical structure among populations of *Ch. utilis*. The ITS sequence shows that total genetic (*H_T_*) and the average within-population diversity (*H_S_*) were 0.868 and 0.495, respectively. The population genetic differentiation coefficients are similar to the cpDNA data (*G_ST_* = 0.430, *N_ST_* = 0.543, *N_ST_* > *G_ST_*, *p* < 0.05). The gene flow based on the two molecular markers was 0.11 and 0.56, respectively.

**Table 5:** The genetic parameters and gene flow of cpDNA and nrDNA.

	*Ht*	*Hs*	*G_ST_*	*N_ST_*	*N_ST_*:*G_ST_*	Nm
cpDNA	0.956	0.507	0.470	0.976	*N_ST_* > *G_ST_*	0.11
nrDNA	0.868	0.495	0.430	0.543	*N_ST_* > *G_ST_*	0.56

**Figure 3:**
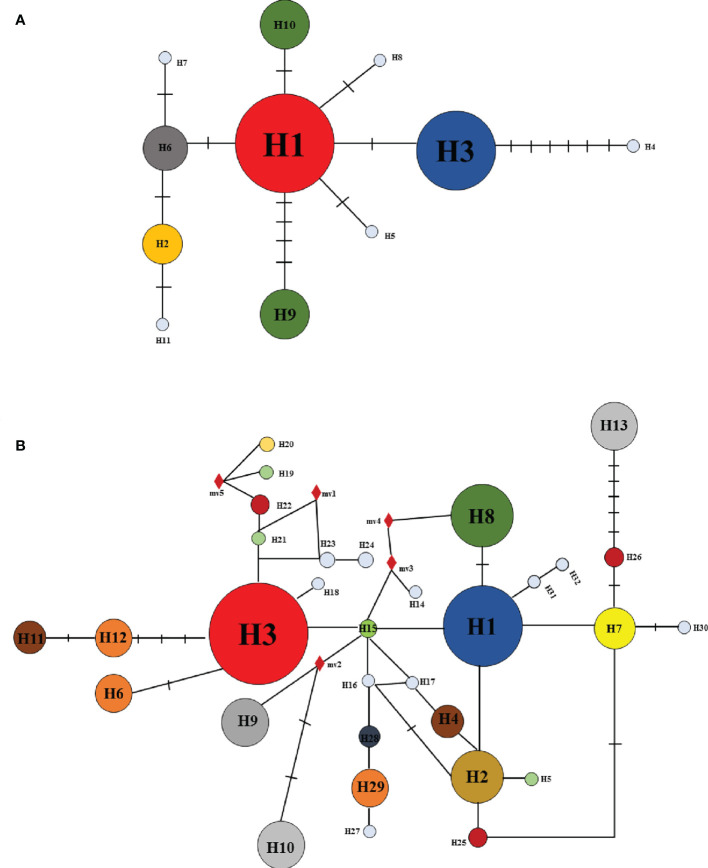
Haplotype relationships based on cpDNA **(A)** and nrDNA **(B)** by the median-joining network.

For combined cpDNA, the H1 haplotype has the widest distribution and the highest frequency, followed by haplotype H3. According to the NETWORK analysis, these two widespread haplotypes are located at the central position of the network diagram and are likely the ancestral haplotypes. The remaining eight haplotypes with low frequency were located at external positions relative to the two main haplotypes; these may be young haplotypes derived from recent differentiation. The ITS sequence haplotype network diagram results supported the congruent cpDNA haplotypes, which showed that the haplotypes H3 and H1 were distributed in the central position and that the remaining haplotypes were distributed in the outside nodes of the network diagram (**Figure 3**).

**Figure 4:**
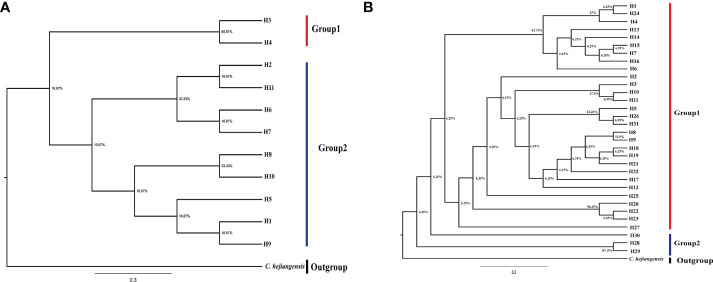
Phylogenetic Tree of *Ch. utilis* based on cpDNA **(A)** and nrDNA **(B)** haplotypes by IQ- tree.

To further understand the phylogenetic analysis of *Ch. utilis*, maximum likelihood analysis was performed for the 11 cpDNA haplotypes and 32 ITS haplotypes with *Chimonobambusa hejiangensis* as an outgroup, with 1,000 bootstrap replications (**Figure 4**). All haplotypes were divided into two clades with low statistical support, possibly due to the short time of intraspecific variations. These results are consistent with the haplotype network diagrams based on cpDNA and ITS sequences.

**Figure 5:**
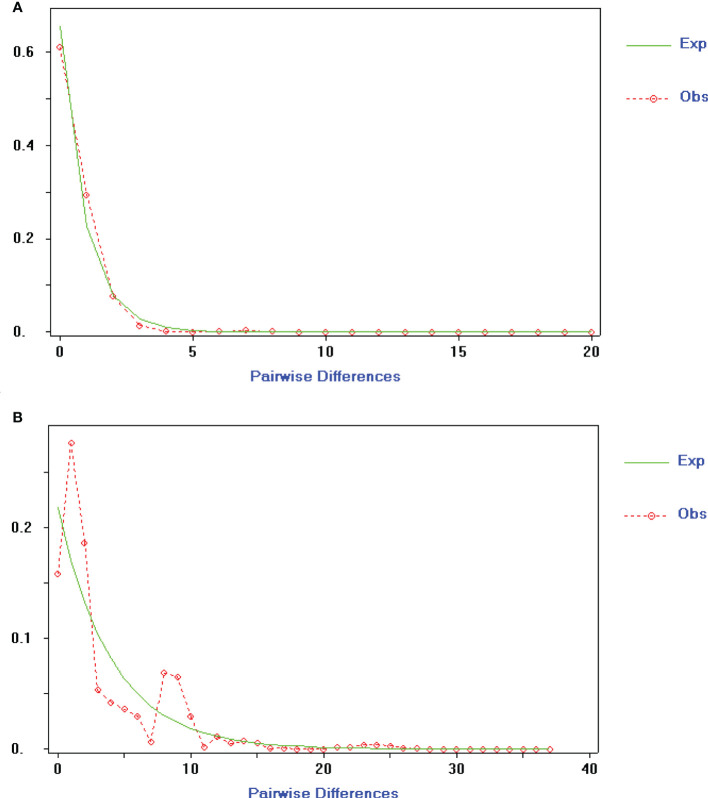
Mismatch-distribution analysis of cpDNA **(A)** and nrDNA **(B)** haplotypes for *Ch. utilis*.

To gain insight into the population and dynamic history of *Ch. utilis*, the mismatch analysis and neutrality test results supported expansions by cpDNA and ITS data. Based on cpDNA combined sequences, the mismatch analysis was unimodal, and the expected value was consistent with the observed value, indicating that this species had recently undergone population expansion. The Neutrality Test showed both significant negative Tajima’s D value (Tajima’s D = −1.60601, 0.10 < *p* < 0.05) and Fu and Li’s F* (Fu and Lis F* = −4.24699, *p* < 0.02), implying that the populations had experienced directional selection or population expansion. The ITS sequence displayed a significant negative Tajima’s D value (Tajima’ s D = −1.76916, *p* < 0.05) and non-significant negative Fu and Li’s F* value (Fu and Li’s F* = −1.80003, *p* > 0.01), whereas the mismatch distribution analysis was bimodal with ITS data (**Figure 5**).

### EST-SSR data analysis

To assess the diversity of *Ch. utilis* populations, five primers were polymorphic. The observed allele number (*Na*) per locus ranged from 3.143 (EST-SSR5) to 4.741 (EST-SSR1), with a mean of 3.848. The effective allele number (*Ne*) per locus ranged from 2.544 (EST-SSR3) to 3.767 (EST-SSR1), with an average value of 3.111. Shannon’s Information Index (*I*) had an average of 0.170 and ranged from 0.991 (EST-SSR3) to 1.373 (EST-SSR1). The observed heterozygosity (*Ho*) and expected heterozygosity (*He*) varied from 0.993 (EST-SSR5) to 1.000 (EST-SSR1–4), and from 0.596 (EST-SSR3) to 0.886 (EST-SSR2), with an average of 0.999 and 0.653, respectively. The value of the polymorphism information content (*PIC*) ranged from 0.592 (EST-SSR3) to 0.886 (EST-SSR2) with a mean of 0.732, showing highly polymorphic loci for five primers. In summary, the highest level of genetic diversity was detected in the locus EST-SSR1, while the lowest level of genetic diversity was detected in the locus EST-SSR3 (**Supplementary Table 5**).

**Table 6** provides the *Ch. utilis* genetic diversity at the population levels. The *Na* and *Ne* per population ranged from 3.000 (CS) to 4.800 (XZ) and from 2.448 (HYS) to 3.714 (QB), with mean values of 3.843 and 3.111, respectively. The *PIC* value shows the genetic diversity of populations; its value ranged from 0.948 (HYS) to 1.346 (YL), with an average of 1.170. The *Ho* value was 1.000 in all populations apart from the QB population (*Ho* = 0.980), with an average of 0.999. The *He* reflected the richness and evenness of the population and varied from 0.584 (HYS) to 0.712 (YL), with an average of 0.653. The genetic diversity of the XZ, QB, YL, and GL populations investigated in the present study was high; however, the genetic diversity of the HYS and CS populations was low. At the population level, the polymorphic loci (PPL) percentage was 100% in all populations (**Table 6**).

**Table 6:** Polymorphism analysis of 14 *Ch. utilis* populations based on EST-SSR.

Population	*Na*	*Ne*	*I*	*Ho*	*He*	PPL (%)
KKS	3.200	2.824	1.046	1.000	0.620	100.00
QB	4.600	3.714	1.289	0.980	0.675	100.00
XF	4.200	3.256	1.225	1.000	0.665	100.00
JS	4.000	3.319	1.261	1.000	0.691	100.00
MT	3.400	2.816	1.111	1.000	0.643	100.00
XY	4.200	3.487	1.242	1.000	0.669	100.00
DSH	3.800	3.210	1.190	1.000	0.664	100.00
XZ	4.800	3.388	1.340	1.000	0.699	100.00
GFD	3.400	2.575	1.010	1.000	0.600	100.00
HYS	3.000	2.448	0.948	1.000	0.584	100.00
GL	4.000	3.302	1.221	1.000	0.669	100.00
JW	3.800	3.028	1.165	1.000	0.653	100.00
YL	4.400	3.633	1.346	1.000	0.712	100.00
CS	3.000	2.558	0.987	1.000	0.602	100.00
Mean	3.843	3.111	1.170	0.999	0.653	100.00

**Figure 6:**
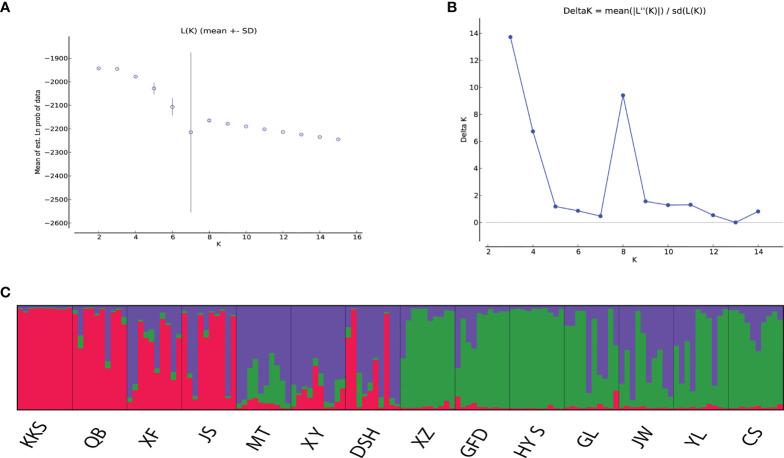
Results of STRUCTURE cluster of 140 individuals of *Ch. utilis*. **(A)** Mean log-likelihood (Ln(K)±S.D.). **(B)** Values of logarithm likelihood based on analysis by structure program. **(C)** Each of color bars represents a kind of genotypic group.

AMOVA based on EST-SSR data among 14 populations of *Ch. utilis* showed that a higher genetic variation was observed within populations (94.98%) than among populations (5.02%), which indicated that the genetic variation mainly occurred within populations. Moreover, the Fixation Index (*F_ST_*) was 0.05021 at the population level (**Supplementary Table 6**). The Bayesian cluster analysis of EST-SSR showed that *K* = 3 was the optimum number of subpopulations, revealing that at least three distinct groups existed among 14 populations. The highest peak (Delta *K* = 13.7167) was at the value *K* = 3. Overall, there were different degrees of introgression among 140 individuals from different populations (**Figure 6**).

**Figure 7:**
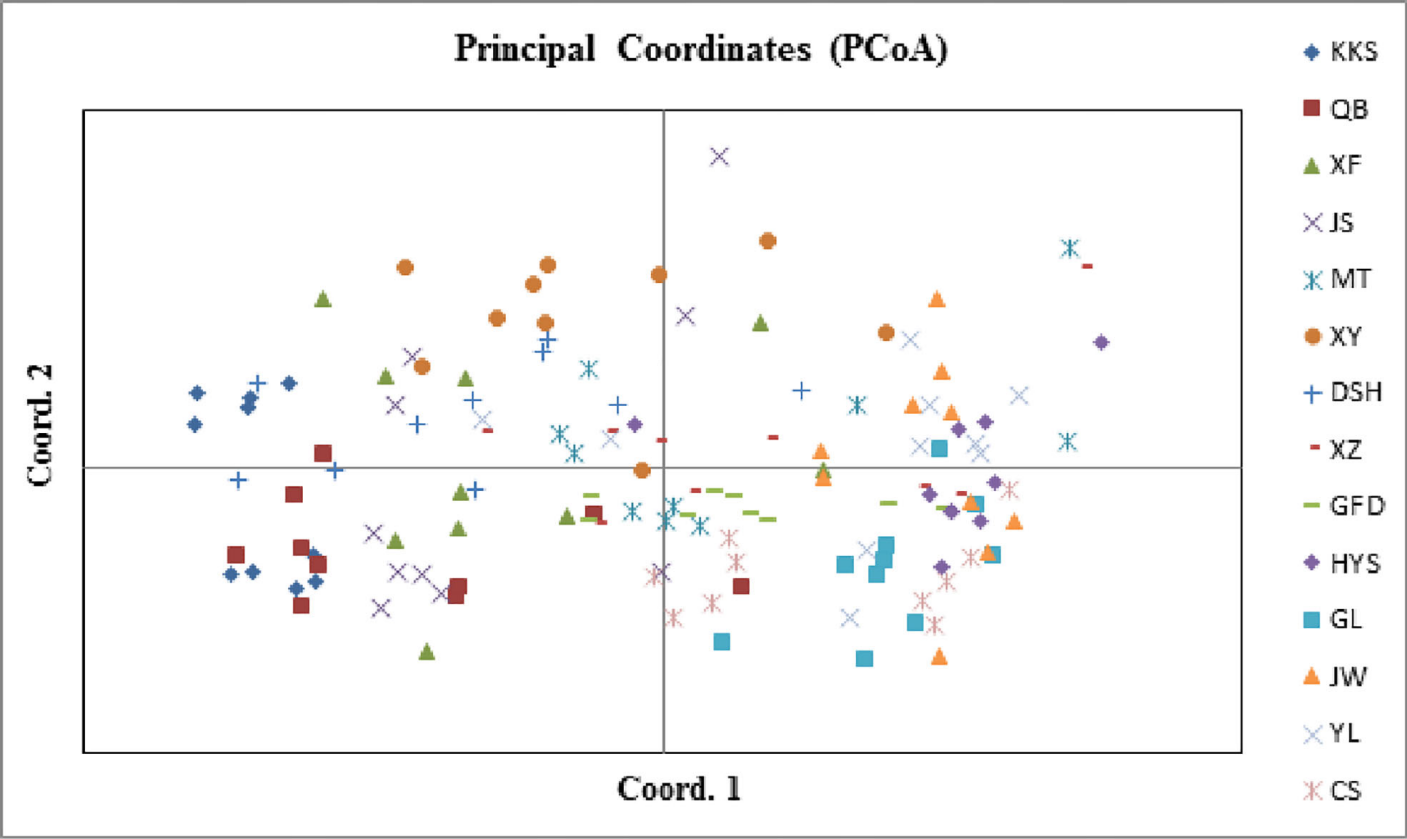
Principal coordinates analysis of *Ch. utilis* individuals.

**Figure 8:**
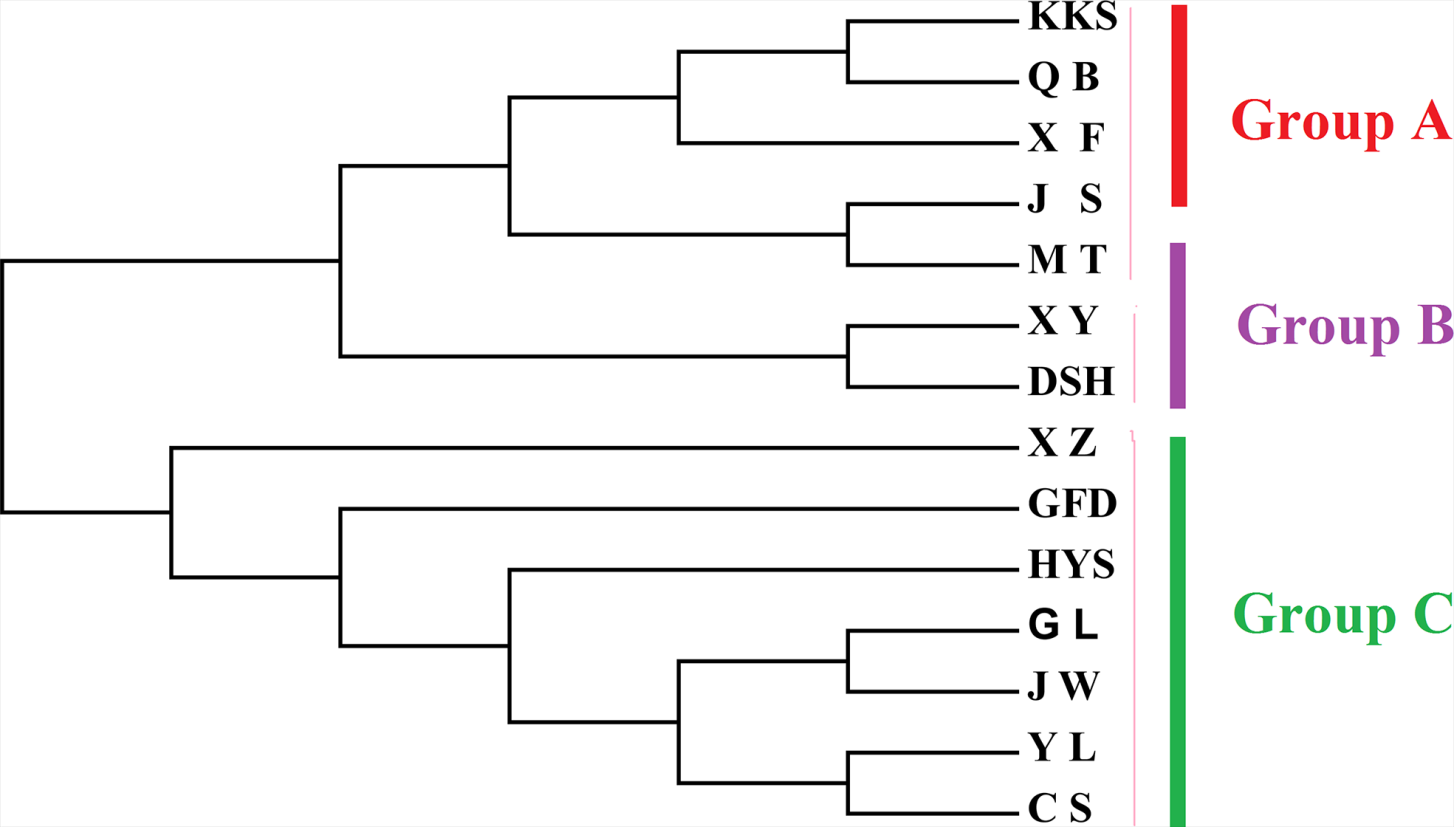
NJ tree of *Ch. utilis* populations based on Nei’s genetic distance.

AUPGMA dendrogram was constructed based on Nei’s genetic distances. PCoA results showed that all individuals were divided into three groups, which are identical to those determined by the UPGMA analysis. The first, second, and third components accounted for 16.01%, 26.67%, and 35.22% of the genetic similarity among all individuals, respectively (**Figure 7**). Combined with geographical distribution, all populations were divided into three sites (**Figure 8**): the southern region (Group A), the northern region (Group B), and the northwestern region (Group B).

**Figure 9:**
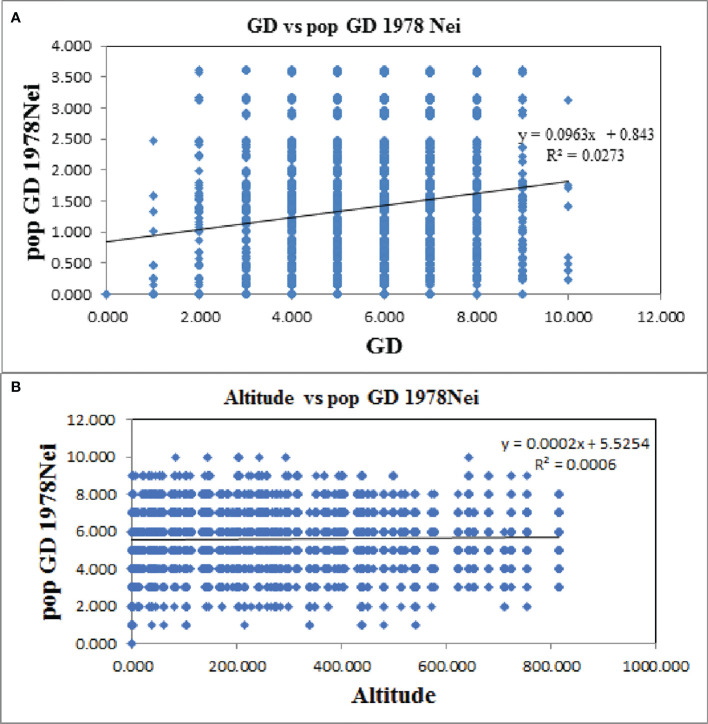
Mantel test between genetic distance **(A)** and geographic distance **(B)** of *Ch. utilis* based on EST-SSR marker.

Furthermore, the Mantel test revealed a significant positive correlation between genetic distance and geographic distance in *Ch. utilis* (*R* ^2^ = 0.0273, *p* = 0.000; **Figure 9A**). However, the Mantel test of genetic distance and altitude showed no significant positive correlation. These results provided further evidence that geographic isolation was the primary factor leading to the genetic differentiation of *Ch. utilis* (*R* ^2^ = 0.0006, *p* = 0.177; **Figure 9B**). Bottleneck analysis indicated that except for populations KKS and CS, the bottleneck effect was not detected in other populations based on Sign tests (*p* < 0.05) under the IAM, TPM, and SMM models. Also, the significant probabilities of Wilcoxon (*p* > 0.05) showed that some populations, such as KKS, QB, JS, XZ, HYS, and CS, experienced a recent bottleneck event (**Supplementary Table 7**).

## Discussion

### Genetic variations and differentiation

Biodiversity is the basis of human survival and is characterized by a complex ecological mixture of all biological species and their living environment. Biodiversity is mainly divided into three levels: species diversity, genetic diversity, and ecosystem diversity. Genetic diversity generally refers to the sum of genetic variation of all individuals in different populations within a species, which has become a key metric in conservation biology research. Studying the origin and evolution of species provides a basis for the rational utilization of species and the formulation of conservation measures. *Ch. utilis* is mainly distributed in the Daluoshan Mountains and is characterized by a narrow distribution. *Camellia huana*, also distributed in the limestone-hilly areas of southern Guizhou and northern Guangxi, is an endangered species. A total of 99 alleles were detected in 12 populations of *C. huana* using 12 pairs of SSR molecular markers, indicating a low level of genetic diversity (Li et al., 2020). The present study detected higher genetic variations than endangered species, such as *Ipomoea cavalcantei* and *Pityopsis ruthii* (Hatmaker et al., 2018; Lanes et al., 2018). However, using cpDNA and ITS markers, the degree of genetic diversity of *Ch. utilis* is low relative to other angiosperms. Generally, the species with wide geographical distribution and large populations have a higher level of genetic diversity and a relatively rich gene pool, so they have a strong ability to adapt to different environments. For *Oxytenanthera abyssinica*, which mainly grows in the African continent (altitude range is 540 m to 1,750 m), the range of nucleotide polymorphism (π) ranged from 0.5 × 10^−3^ to 6.3 × 10^−3^ based on cpDNA fragment analysis (Oumer et al., 2021), which is higher than that of *Ch. utilis* in this study. In addition, there is little gene flow and high genetic differentiation among long-distance populations of *O. abyssinica*, which is similar to the results of this study. We speculate that this is mainly a result of geographical obstacles.

In this study, there are great differences in genetic diversity among different populations of *Ch. utilis*. Combined with the geographical distribution map, populations MB and DSH in the north and populations JS, GL, and CS in the southwest of the Daluoshan Mountains have high haplotype polymorphism and nucleotide polymorphism. Genetic diversity is an important indicator to measure the degree of variation in a population. The higher the degree of the population, the higher the genetic diversity and richer genetic resources, which helps to improve the adaptability of the species to climate change and historical events and provides a theoretical basis for screening for good populations.

### Relationships and structure in the *Ch. utilis* populations

Bambusoideae is the only herbaceous plant with lignification under Poaceae. Bamboo has strong adaptability to the environment and easily forms varieties because of different habitats, genetic drift, human transplanting, and other factors. In addition, China has a vast territory and complex topographic and climatic changes. Incomplete statistics show that there are more than 100 varieties or forms of bamboo in China (Qiu et al., 2001). This complicates the traditional classification methods, which are mainly based on the morphological characteristics of flowers and fruits. Therefore, the study on the genetic diversity of bamboos can provide a molecular basis for controversial morphological classification results.

Barkley et al. (2005) used EST-SSR molecular markers to detect the genetic variation among 92 individuals of 44 bamboo species in 11 genera, and the UPGMA clustering method was used to classify these bamboo species into two categories, which was consistent with the morphological classification according to the different sympodial and monopodial. Mukherjee et al. (2010) used four pairs of EST-SSR primers to carry out UPGMA and SAHN cluster analysis of 20 bamboo species growing in India. It was found that the systematic location of a few bamboo species was not consistent with the morphological classification results, forming other evolutionary branches, requiring further detailed taxonomic study.

In this study, based on the structural analysis results, 14 populations of *Ch. utilis* could be divided into three groups according to the most suitable K value. According to their geographical distribution, all populations could be divided into southern, northern, and western regions along the Daluoshan Mountains. The results of the NJ tree and PCoA analysis based on Nei’s genetic distance supported the division of *Ch. utilis* into three groups. Southwest China has a varied terrain, as well as a great diversity of climates; therefore, the general division of *Ch. utilis* populations into three groups may be affected by its own environmental tolerance, diffusion capacity, geological events, and climate fluctuations.

CpDNA data showed that the *G_ST_* of the *Ch. utilis* population in *Ch. utilis* was lower than the average *G_ST_* (*G_ST_* = 0.470) of the other 124 angiosperms (by 0.637). However, compared with the above cpDNA data, the results based on nrDNA analysis showed that the *G_ST_* of *Ch. utilis* was higher than the average *G_ST_* (*G_ST_* = 0.430) of 77 angiosperms by 0.184 (Petit et al., 2005). Liu et al. (2012) analyzed the genetic structure of 21 natural populations of *Camellia taliensis* using cpDNA gene fragments and ITS sequences. These authors found that, based on cpDNA data, the degree of genetic differentiation among *Camellia taliensis* populations (*G_ST_* = 0.988, *N_ST_* = 0.989) was higher than that of the *Ch. utilis* population (*G_ST_* = 0.470, *N_ST_* = 0.976). This may be because the effective population size of the organelle DNA of *C. taliensis* is smaller than that of nuclear DNA, resulting in strong genetic drift and a higher level of population differentiation. However, the degree of genetic differentiation among populations of *Ch. utilis* was higher than that of some conifers (Hamrick and Godt, 1996; Petit et al., 2005).

In this study, data analysis based on cpDNA and nrDNA showed that chloroplast haplotype H1 and ribosomal haplotype H3 are at the center of the haplotype network diagram, respectively, with a large proportion and the most widespread distribution. Therefore, we speculate that haplotypes H1 and H3 are ancient haplotypes of the *Ch. utilis* population. In addition, the haplotype phylogenetic tree based on cpDNA data divides all groups into two groups. According to the geographical distribution characteristics, the southwest side of the Daloushan Mountains is blocked by the Yunnan-Guizhou Plateau, and there are Wuling Mountains in the northeast. Therefore, populations DSH, GL, ZX, and XF in these two locations of the Daluoshan Mountains contain stable and unique haplotypes H11, H4, H5, and H9, respectively. Its haplotype diversity and nucleotide diversity are also high, and it is geographically far away from the Daluoshan Mountains. Rich haplotypes and genetic diversity allow post-glacial species to re-expand from glacial sanctuaries. Therefore, based on further analysis of nrDNA data, this study speculated that there were at least two *Ch. utilis* population refuges during the glacial period.

### Management and protection strategy

Maintaining population genetic diversity and intraspecific genetic variation is not only a source of biological evolution but also a prerequisite for the sustainable development of this species and an important part of biodiversity conservation (Rao, 2004).

Based on the distribution characteristics of the *Ch. utilis* populations and the significant genetic differentiation among populations, it is necessary to protect the existing populations through *in situ* and *ex situ* conservation efforts. This study showed that the genetic diversity of *Ch. utilis* populations was highest in the north and southwest Daloushan Mountains. In the EST-SSR analysis, XZ, QB, YL, GL, and other populations in these two regions had a high level of genetic diversity. The cpDNA and ITS sequence analyses detected many haplotypes and endemic haplotypes in these two regions, and they have high haplotype polymorphism and nucleotide polymorphism. Therefore, to effectively protect the genetic germplasm resources of *Ch. utilis* in these two areas, especially the populations that have recently experienced bottleneck effects (such as HYS and CS populations), we should implement conservation *in situ* strategies to maintain the existing population size, avoid the loss of haplotypes caused by environmental changes, and improve the adaptability of the population. At the same time, special haplotype populations are transplanted to places with similar environments, and conservation *ex situ* strategies are implemented. For example, the construction of seed resources or nature reserves, such as the established Dashahe Nature Reserve and Kuanshui Nature Reserve, improves the survival rate of cultivation and improves management.

## Conclusion

In this study, 20 populations of *Ch. utilis* were collected, including 13 populations in Guizhou Province, two populations in Chongqing, two populations in Yunnan Province, and three populations in Sichuan Province. Five pairs of EST-SSR molecular markers were used to analyze the genetic diversity, genetic structure, and bottleneck effect of 14 populations. Three cpDNA fragments (*trn*H-*psb*A, *atp*F-*atp*H, and *psb*K-*psb*I) with uniparental inheritance and one ITS sequence with biparental inheritance were used to study the genetic diversity, genetic structure, and dynamic population history of 410 individuals from 20 populations of *Ch. utilis*. We detected a very significant positive correlation between genetic distance and geographical distance among populations, indicating that the genetic variation among populations of *Ch. utilis* is mainly affected by geographical barriers. In addition, the neutral test results of the two markers were significantly negative, indicating that the population of *Ch. utilis* underwent a recent rapid expansion event. Based on the present population distribution, genetic diversity, and geographical pedigree structure of *Ch. utilis*, a scientific and feasible strategy for protection and utilization was put forward. This study aimed to provide a theoretical basis for an in-depth understanding of the population genetic structure, germplasm sampling, geological events, and climate change on the genetic differentiation and geographical distribution pattern formation mechanism of *Ch. utilis*.

## Data availability statement

The datasets presented in this study can be found in online repositories. The names of the repository/repositories and accession number(s) can be found in the article/**Supplementary Material**.

## Author contributions

YL performed the experiments, analyzed the data, and approved the final draft. MW contributed to the investigation. XX and ZX prepared figures and/or tables. ZD and GG conceived and designed the experiments. All authors agree to be accountable for the final manuscript. All authors contributed to the article and approved the submitted version.

## Funding

This work was supported by the Breeding of Improved Bamboo Varieties for Shoot (Te-Lin-Yan 2020-17).

## Acknowledgments

We thank the Investigation of Bamboo Resources and Excavation of Economic Bamboo Species in Guizhou Province.

## Conflict of interest

The authors declare that the research was conducted in the absence of any commercial or financial relationships that could be construed as a potential conflict of interest.

## Publisher’s note

All claims expressed in this article are solely those of the authors and do not necessarily represent those of their affiliated organizations, or those of the publisher, the editors and the reviewers. Any product that may be evaluated in this article, or claim that may be made by its manufacturer, is not guaranteed or endorsed by the publisher.
