# Supplementary material for: Genetic diversity and phylogeography of the endemic species Chimonobambusa utilis growing in southwest China: Chloroplast DNA sequence and microsatellite marker analyses
Source: Front Plant Sci. 2022 Nov 3;13:943225. doi: 10.3389/fpls.2022.943225 (PMC9671600; doi:10.3389/fpls.2022.943225)
Supplement: Supplementary file 1 [file Table_1.docx]

Supplementary Table 1 Sampling information of *Ch. utilis* based on EST-SSR.

| **ID** | **Location** | **Individuals (n)** | **Latitude (N)** | **Longitude (E)** | **Altitude (m)** |
| --- | --- | --- | --- | --- | --- |
| KKS | Kuankuoshui, GuiZhou | 20 | 107.17 | 28.20 | 1433 |
| QB | Qingba, GuiZhou | 20 | 107.04 | 28.26 | 1781 |
| XF | Fengcisi, GuiZhou | 20 | 106.63 | 28.30 | 1431 |
| JS | Taiping, GuiZhou | 20 | 105.90 | 27.13 | 1324.43 |
| XY | Xianyuan, GuiZhou | 20 | 106.70 | 28.78 | 1633.75 |
| LSG | Loushanguan, GuiZhou | 20 | 106.83 | 28.58 | 1724.86 |
| DSH | Dashahe, GuiZhou | 20 | 107.58 | 28.57 | 1351.06 |
| XZ | XinZhou, GuiZhou | 20 | 107.25 | 28.78 | 1394.94 |
| GFD | Gufodong, ChongQing | 20 | 107.18 | 29.03 | 2074.47 |
| HYS | Huayingshan, SiChuan | 20 | 106.79 | 30.30 | 1972.11 |
| GL | Gulin, SiChuan | 20 | 105.68 | 28.10 | 1533.77 |
| JW | Xuyong, SiChuan | 20 | 105.59 | 28.21 | 1320.04 |
| ZX | Zhenxiong, YunNan | 25 | 104.33 | 27.66 | 1799.96 |
| CS | Liangkouhe, GuiZhou | 22 | 105.71 | 28.35 | 1260.49 |
