# Supplementary material for: Genetic diversity and phylogeography of the endemic species Chimonobambusa utilis growing in southwest China: Chloroplast DNA sequence and microsatellite marker analyses
Source: Front Plant Sci. 2022 Nov 3;13:943225. doi: 10.3389/fpls.2022.943225 (PMC9671600; doi:10.3389/fpls.2022.943225)
Supplement: Supplementary file 2 [file Table_2.docx]

Supplementary Table 2 Screening for qualified cpDNA and nrDNA primer pair sequences

| Primers | Sequence（5′–3′） | Tm (℃) | References |
| --- | --- | --- | --- |
| trnH-psbA | CGCGCATGGTGGATTCACAAT CC | 55 | Cai ZM et al., 2012 |
|  | GTTATGCATGAACGTAATGCTC |  |  |
| atpF-atpH | ACTCGCACACACTCCCTTTCC |  |  |
|  | GCTTTTATGGAAGCTTTAACAAT |  |  |
| psbK-psbI | TTAGCCTTTGTTTGGCAAG |  |  |
|  | AGAGTTTGAGAGTAAGCAT |  |  |
| ITS5- ITS4 | GGAAGTAAAAGTCGTAACAAGG |  |  |
|  | TCCTCCGCTTATTGATATGC |  |  |
