# Supplementary material for: Genetic diversity and phylogeography of the endemic species Chimonobambusa utilis growing in southwest China: Chloroplast DNA sequence and microsatellite marker analyses
Source: Front Plant Sci. 2022 Nov 3;13:943225. doi: 10.3389/fpls.2022.943225 (PMC9671600; doi:10.3389/fpls.2022.943225)
Supplement: Supplementary file 3 [file Table_3.docx]

| Primer | Sequence (5′–3′) | Reference | Tm (℃) |
| --- | --- | --- | --- |
| EST-SSR1 | ATTAGACCCTGGGATGGTCC | Zhu et al., 2021 | 60.24 |
|  | GCGGTAAGAGAGGTTACCCA |  |  |
| EST-SSR2 | ATGCTGACAAGGGATAACCG |  | 60.0 |
|  | ATTTCAACAAACTCGCCTGC |  |  |
| EST-SSR3 | TCTGATTTGTTTACGCACGC |  | 60.2 |
|  | TCACTGAAACTGGGCATCAA |  |  |
| EST-SSR4 | CCCCACTTCTCTCTCGTTTG |  | 60 |
|  | GCCGCGCAGTAAATAAGAAG |  |  |
| EST-SSR5 | TGTGATCATGTTCATCGAGTT | Li, 2008 | 55.2 |
|  | TTGTTTACCACTTCCTTTGC |  |  |

Supplementary Table 3 Screening for qualified EST-SSR primer pair sequences
