# Supplementary material for: Genetic diversity and phylogeography of the endemic species Chimonobambusa utilis growing in southwest China: Chloroplast DNA sequence and microsatellite marker analyses
Source: Front Plant Sci. 2022 Nov 3;13:943225. doi: 10.3389/fpls.2022.943225 (PMC9671600; doi:10.3389/fpls.2022.943225)
Supplement: Supplementary file 4 [file Table_4.docx]

Supplementary Table 4 Summary of polymorphisms in cpDNA sequences

| Haplotype | *trnH-psbA* | | *atpF-atpH* | | | | | | | | | | *psbK-psbI* | | | | | | | |
| --- | --- | --- | --- | --- | --- | --- | --- | --- | --- | --- | --- | --- | --- | --- | --- | --- | --- | --- | --- | --- |
|  | 1  7  0 | 3  1  4 | 4  8  9 | 5  0  0 | 6  2  3 | 6  2  4 | 6  3  9 | 7  2  1 | 8  6  5 | 9  3  9 | 1  0  4  4 | 1  0  4  5 | 1  1  1  5 | 1  1  9  3 | 1  1  9  4 | 1  1  9  5 | 1  1  9  6 | 1  1  9  7 | 1  1  9  8 | 1  3  8  2 |
| H1 | C | A | C | T | A | A | A | T | A | - | A | A | T | - | - | - | - | - | - | C |
| H2 | C | A | C | T | T | A | A | T | A | - | A | A | A | - | - | - | - | - | - | C |
| H3 | C | G | C | T | A | A | A | T | A | - | A | A | T | - | - | - | - | - | - | C |
| H4 | C | G | G | G | A | G | G | G | A | - | G | A | T | - | - | - | - | - | - | C |
| H5 | C | A | C | T | A | A | A | T | A | T | A | A | T | - | - | - | - | - | - | C |
| H6 | C | A | C | T | A | A | A | T | A | - | A | A | A | - | - | - | - | - | - | C |
| H7 | C | A | C | T | A | A | A | T | - | - | A | A | A | - | - | - | - | - | - | C |
| H8 | C | A | C | T | A | A | A | T | A | - | A | G | T | - | - | - | - | - | - | C |
| H9 | C | A | C | T | A | A | A | T | A | - | A | A | T | C | C | C | A | C | C | C |
| H10 | C | A | C | T | A | A | A | T | A | - | A | A | T | - | - | - | - | - | - | T |
| H11 | A | A | C | T | T | A | A | T | A | - | A | A | A | - | - | - | - | - | - | C |
