# Supplementary material for: Genetic diversity and phylogeography of the endemic species Chimonobambusa utilis growing in southwest China: Chloroplast DNA sequence and microsatellite marker analyses
Source: Front Plant Sci. 2022 Nov 3;13:943225. doi: 10.3389/fpls.2022.943225 (PMC9671600; doi:10.3389/fpls.2022.943225)
Supplement: Supplementary file 5 [file Table_5.docx]

**S**upplementary Table 5 Polymorphism analysis of EST-SSR primers

| Locus | *Na* | *Ne* | *I* | *Ho* | *He* | *Hs* | *H_T_* | *PIC* | *Fis* | *Fit* | *Fst* |
| --- | --- | --- | --- | --- | --- | --- | --- | --- | --- | --- | --- |
| EST-SSR1 | 4.741 | 3.767 | 1.373 | 1.000 | 0.713 | 0.704 | 0.858 | 0.843 | -0.403 | -0.166 | 0.169 |
| EST-SSR2 | 4.571 | 3.762 | 1.365 | 1.000 | 0.711 | 0.678 | 0.844 | 0.886 | -0.407 | -0.114 | 0.208 |
| EST-SSR3 | 3.214 | 2.544 | 0.991 | 1.000 | 0.596 | 0.596 | 0.656 | 0.592 | -0.679 | -0.525 | 0.091 |
| EST-SSR4 | 3.571 | 2.790 | 1.093 | 1.000 | 0.629 | 0.629 | 0.687 | 0.645 | -0.589 | -0.426 | 0.102 |
| EST-SSR5 | 3.143 | 2.693 | 1.029 | 0.993 | 0.617 | 0.597 | 0.715 | 0.696 | -0.608 | -0.355 | 0.157 |
| Mean | 3.848 | 3.111 | 0.170 | 0.999 | 0.653 | 0.364 | 0.752 | 0.732 | -0.537 | -0.317 | 0.146 |

Note: *Na*, Number of alleles; *Ne*, Effective number of alleles; *I*, Shannon's Information Index; *Ho*, Observed heterozygosity; *He*, Expected heterozygosity; Polymorphism information content; *Fis*, Inbreeding coefficient at the population level; *Fit*, Inbreeding coefficient at total population level; *Fst*, Proportion of differentiation among populations.
