# Supplementary material for: Genetic diversity and phylogeography of the endemic species Chimonobambusa utilis growing in southwest China: Chloroplast DNA sequence and microsatellite marker analyses
Source: Front Plant Sci. 2022 Nov 3;13:943225. doi: 10.3389/fpls.2022.943225 (PMC9671600; doi:10.3389/fpls.2022.943225)
Supplement: Supplementary file 6 [file Table_6.docx]

**Supplementary Table 6 AMOVA analysis of 14 populations of *Ch. utilis* by EST-SSR**

| Source of variation | d.f. | Sum of squares | Variance components | Percentage of variation (%) | Fixation Index  (*F_st_*) |
| --- | --- | --- | --- | --- | --- |
| Among populations | 13 | 8.386 | 0.01658 | 5.02 | 0.05021 |
| Within populations | 266 | 83.400 | 0.31353 | 94.98 |  |
| Total | 279 | 91.786 | 0.33011 |  |  |
