# Supplementary material for: Genetic diversity and phylogeography of the endemic species Chimonobambusa utilis growing in southwest China: Chloroplast DNA sequence and microsatellite marker analyses
Source: Front Plant Sci. 2022 Nov 3;13:943225. doi: 10.3389/fpls.2022.943225 (PMC9671600; doi:10.3389/fpls.2022.943225)
Supplement: Supplementary file 7 [file Table_7.docx]

| Pop. | IAM | | TPM | | SMM | |
| --- | --- | --- | --- | --- | --- | --- |
|  | Sign test | Wilcoxon test | Sign test | Wilcoxon test | Sign test | Wilcoxon test |
| KKS | 0.045874* | 0.015625* | 0.058047 | 0.015625* | 0.065556 | 0.015625* |
| QB | 0.273313 | 0.031250* | 0.347181 | 0.312500 | 0.377267 | 0.046875* |
| XF | 0.285256 | 0.046875* | 0.600435 | 0.500000 | 0.620427 | 0.109375 |
| JS | 0.283904 | 0.031250* | 0.338204 | 0.109375 | 0.397217 | 0.031250* |
| MT | 0.284143 | 0.312500 | 0.683272 | 0.500000 | 0.642526 | 0.406250 |
| XY | 0.360902 | 0.312500 | 0.298130 | 0.406250 | 0.665423 | 0.312500 |
| DSH | 0.293158 | 0.109375 | 0.303283 | 0.312500 | 0.348717 | 0.312500 |
| XZ | 0.318886 | 0.031250* | 0.321865 | 0.406250 | 0.652166 | 0.046875* |
| GFD | 0.276777 | 0.078125 | 0.659915 | 0.593750 | 0.677441 | 0.406250 |
| HYS | 0.058050 | 0.015625* | 0.288320 | 0.109375 | 0.343300 | 0.031250* |
| GL | 0.584280 | 0.109375 | 0.654972 | 0.687500 | 0.662832 | 0.500000 |
| JW | 0.237017 | 0.031250* | 0.258975 | 0.312500 | 0.307186 | 0.078125 |
| YL | 0.297279 | 0.046875* | 0.308295 | 0.406250 | 0.675376 | 0.109375 |
| CS | 0.049741* | 0.015625* | 0.280079 | 0.078125 | 0.365681 | 0.031250* |

Supplementary Table 7 Bottleneck effect analysis of 14 *Ch. utilis* populations based on three mode.

Note: * indicate P < 0.05.
